# Effectiveness of One-Year Pemafibrate Therapy on Non-Alcoholic Fatty Liver Disease Refractory to Long-Term Sodium Glucose Cotransporter-2 Inhibitor Therapy: A Pilot Study

**DOI:** 10.3390/life13061327

**Published:** 2023-06-05

**Authors:** Satoshi Shinozaki, Toshiyuki Tahara, Kouichi Miura, Alan Kawarai Lefor, Hironori Yamamoto

**Affiliations:** 1 Shinozaki Medical Clinic, Utsunomiya 321-3223, Japan; shinozaki-s@aqua.ocn.ne.jp; 2 Department of Medicine, Division of Gastroenterology, Jichi Medical University, Tochigi 329-0431, Japan; 3 Saiseikai Utsunomiya Hospital, 911-1 Takebayashi, Utsunomiya 321-0974, Japan; 4 Department of Surgery, Jichi Medical University, Tochigi 329-0431, Japan

**Keywords:** non-alcoholic fatty liver disease, non-alcoholic steatohepatitis, pemafibrate, dyslipidemias, PPAR-alpha, sodium glucose transporter-2 inhibitors

## Abstract

**Background:** Both pemafibrate and sodium glucose cotransporter-2 (SGLT2) inhibitor can decrease serum transaminase levels in patients with non-alcoholic fatty liver disease (NAFLD) complicated with dyslipidemia and type 2 diabetes mellitus (T2DM), respectively. However, the effectiveness of combined therapy has been rarely reported. **Methods:** This is a two-center retrospective observational study. NAFLD patients complicated with T2DM treated with pemafibrate for >1 year were included, in whom prior treatment with SGLT2 inhibitor > 1 year failed to normalize serum alanine aminotransferase (ALT) levels. Hepatic inflammation, function, and fibrosis were assessed by ALT, albumin-bilirubin (ALBI) score, and Mac-2 binding protein glycosylation isomer (M2BPGi) levels, respectively. **Results:** Seven patients were included. The median duration of prior treatment with SGLT2 inhibitors was 2.3 years. During the one year before starting pemafibrate therapy, the therapy did not significantly change hepatic enzymes. All patients received pemafibrate 0.1 mg twice daily without dose escalations. During one year of pemafibrate therapy, triglyceride, aspartate aminotransferase, ALT, γ-glutamyl transpeptidase, ALBI score, and M2BPGi levels significantly improved (*p* < 0.05), although weight or hemoglobin A1c did not significantly change. **Conclusions:** One year of pemafibrate therapy improves markers of hepatic inflammation, function, and fibrosis in NAFLD patients in whom long-term SGLT2 inhibitor therapy failed to normalize serum ALT.

## 1. Introduction

Non-alcoholic fatty liver disease (NAFLD) is one of the most common liver diseases. The worldwide prevalence is estimated at 32.4% and still increasing even in developing countries [1,2]. The growing number of patients with NAFLD will have serious economic and medical consequences. Indeed, NAFLD is associated with increased all-cause mortality, including liver-related mortality due to hepatic failure and hepatocellular carcinoma [3]. Thus, long-term control of hepatic inflammation, function, and fibrosis associated with NAFLD is important to improve the prognosis because NAFLD can progress to cirrhosis and develop hepatocellular carcinoma [4].

Currently, no treatment agents are approved for NAFLD. The incidence of type 2 diabetes mellitus (T2DM) is closely associated with NAFLD [5] because NAFLD is related to insulin resistance and the development of metabolic syndrome. Sodium glucose cotransporter-2 (SGLT2) inhibitors have been used for patients with T2DM, in whom the urinary excretion of glucose is promoted. In addition, SGLT2 inhibitors are reported to improve steatosis and fibrosis in patients with both NAFLD and T2DM [6]. We previously reported that one year of treatment with an SGLT2 inhibitor provided favorable outcomes in patients with NAFLD and T2DM [7]. However, normalization of alanine aminotransferase (ALT) (≤30 U/L) was accomplished in 58% (14/24) of patients, and therefore 42% were considered refractory to one year of SGLT2 inhibitor therapy [7]. In addition, treatment with an SGLT2 inhibitor did not decrease serum lipid levels, including triglyceride [7].

Dyslipidemia is also associated with NAFLD. Pemafibrate, a novel selective peroxisome proliferator-activated receptor (PPAR)-alpha modulator, is approved for patients with dyslipidemia. In a recent randomized controlled trial (RCT) using pemafibrate for 10,479 patients with T2DM and mild-to-moderate hypertriglyceridemia, pemafibrate significantly reduced the incidence of NAFLD [8]. As expected, pemafibrate therapy decreased serum triglyceride, very-low-density lipoprotein (VLDL) cholesterol, and remnant cholesterol levels, compared to the placebo group at four months. In accordance with improved metabolic parameters, a lower incidence of NAFLD was shown compared to the placebo group (hazard ratio, 0.78; 95% confidence interval, 0.63 to 0.96) [8]. All three of our studies employing short-, mid-, and long-term pemafibrate therapy supported the beneficial effects of pemafibrate on patients with NAFLD [9,10,11]. In the short-term study, 28 patients without T2DM treated with pemafibrate for three months had a significant decrease in ALT and increase in serum albumin, high-density lipoprotein (HDL) cholesterol, and platelet count [9]. In the mid-term study including 71 patients without T2DM, pemafibrate therapy improved hepatic inflammation and fibrosis. Of note, these beneficial effects were more evident in lean NAFLD (body mass index [BMI] < 25) than obese NAFLD (BMI > 30) [11]. In the long-term study including 22 patients without T2DM, sustained improvements in hepatic inflammation, function, and fibrosis were demonstrated during the one-year pemafibrate therapy [10]. Thus, the beneficial effects of pemafibrate on NAFLD is evident.

One of the remaining issues is NAFLD patients with T2DM who failed to normalize serum ALT levels by SGLT2 inhibitors, herein defined as “refractory to SGLT2 inhibitor”. Based on a published report [8] and our previous data [9,10,11], we believed that pemafibrate therapy may be effective for NAFLD patients refractory to SGLT2 inhibitors because these two agents have different mechanisms to improve serum ALT levels. Although the combination therapy with pemafibrate and SGLT2 inhibitor was reported in an animal study [12], few clinical data were available. The aim of this study was to investigate the long-term effectiveness of the combined therapy with pemafibrate and SGLT2 inhibitor for NAFLD patients who were “refractory to long-term SGLT2 inhibitor”.

## 2. Methods

### 2.1. Study Population

This is a two-center retrospective observational study including consecutive patients with NAFLD treated with pemafibrate begun after a course of SGLT2 inhibitor therapy between June 2019 and March 2021 at the Saiseikai Utsunomiya Hospital and the Shinozaki Medical Clinic. We defined “refractory to SGLT2 inhibitor therapy” as sustained ALT elevation > 30 U/L for more than three months before starting pemafibrate therapy. We included the patients who were refractory to SGLT2 inhibitor therapy for more than one year and underwent continuous pemafibrate therapy. All patients included were diagnosed as having fatty liver by abdominal ultrasound and had dyslipidemia. Normal serum IgG level, negative hepatitis B surface antigen, or anti-hepatitis C antibody were confirmed. Based on the diagnostic criteria of NAFLD, their alcohol consumption was <30 g/day in males and <20 g/day in females [9,10]. We excluded the patients who had severe chronic kidney disease (estimated glomerular filtration rate < 30 mL/min/1.73 m^2^), discontinued pemafibrate or SGLT2 inhibitor therapy within one year, lost to follow-up within one year, or had a history of previous pemafibrate use. This retrospective observational study was approved by the Institutional Review Board at both institutions (ID#2019-56 at the Saiseikai Utsunomiya Hospital and ID#31-R002 at the Shinozaki Medical Clinic).

### 2.2. Markers of Hepatic Inflammation, Function, and Fibrosis

Hepatic inflammation was assessed by serum ALT levels [13,14]. The ALT level is useful to speculate about histological changes associated with NAFLD [14]. Hepatic function was assessed by the albumin-bilirubin (ALBI) score, calculated using serum albumin and total bilirubin levels, which is proportionate to the indocyanine green retention test at 15 min [15]. Mac-2 binding protein glycosylation isomer (M2BPGi) was used to evaluate the grade of hepatic fibrosis because the area under the receiver operating characteristic curve of M2BPGi is superior to other fibrosis markers and scoring systems, including platelet count, hyaluronic acid, aspartate aminotransferase (AST)/ALT ratio, AST-to-platelet ratio index, FIB-4 index, and NAFLD fibrosis score [16].

### 2.3. Statistical Analysis

Changes in parameters from baseline to one year were assessed with the Wilcoxon rank sum test. Comparisons of multiple timing in parameters were evaluated using Friedman’s test. StatFlex 7.0 software (Artech Co., Ltd., Osaka, Japan) was used for all statistical analyses. Differences were considered significant with *p* < 0.05.

## 3. Results

### 3.1. Baseline Characteristics

**Figure 1:**
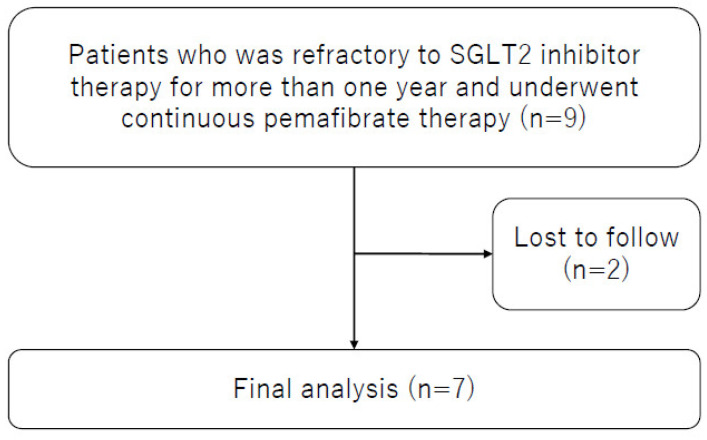
Study flowchart.

Nine patients fulfilled the inclusion criteria, and subsequently two patients were excluded based on the exclusion criteria (Figure 1). The remaining seven patients were included in the final analysis (Table 1). Median treatment duration with an SGLT2 inhibitor (empagliflozin [*n* = 6] and dapagliflozin [*n* = 1]) before starting pemafibrate was 2.3 years. All patients were treated with the addition of pemafibrate 0.1 mg twice daily without dose escalations.

### 3.2. Changes in Parameters during One Year of Pemafibrate Therapy

During one year of pemafibrate therapy, significant improvements were observed in AST, ALT, γ-glutamyl transpeptidase (γ-GTP), triglyceride, total bilirubin, serum albumin, ALBI score, and M2BPGi levels without a significant change in weight (Table 2). Diabetic markers including fasting plasma glucose, serum insulin, and hemoglobin A1c did not change significantly during the treatment period. Although ALT significantly decreased in all patients analyzed, three of seven patients (43%) showed normalization (≤30 IU/L) of serum ALT levels. Additional pemafibrate therapy significantly improved markers of hepatic inflammation (ALT), function (ALBI score), and fibrosis (M2BPGi). No adverse events were observed.

### 3.3. Liver Enzyme Profiles One Year before and One Year after Starting Pemafibrate Therapy

**Figure 2:**
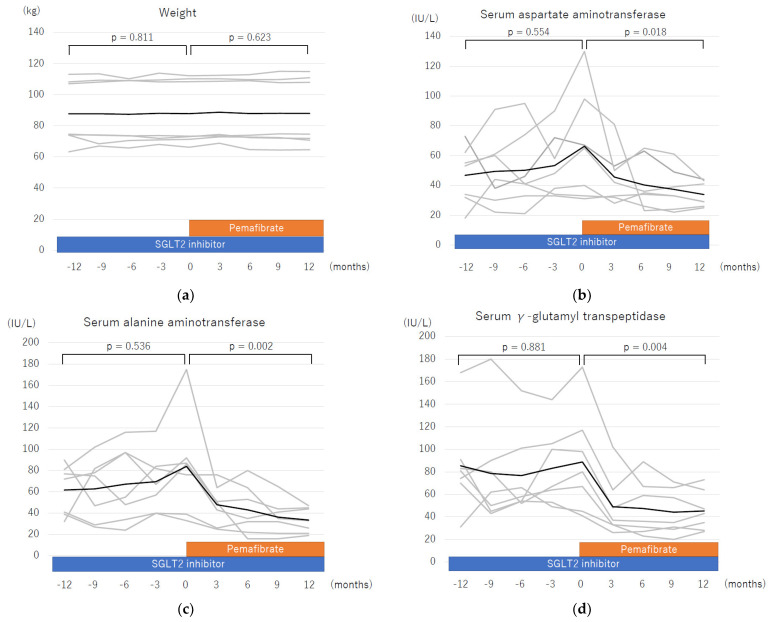

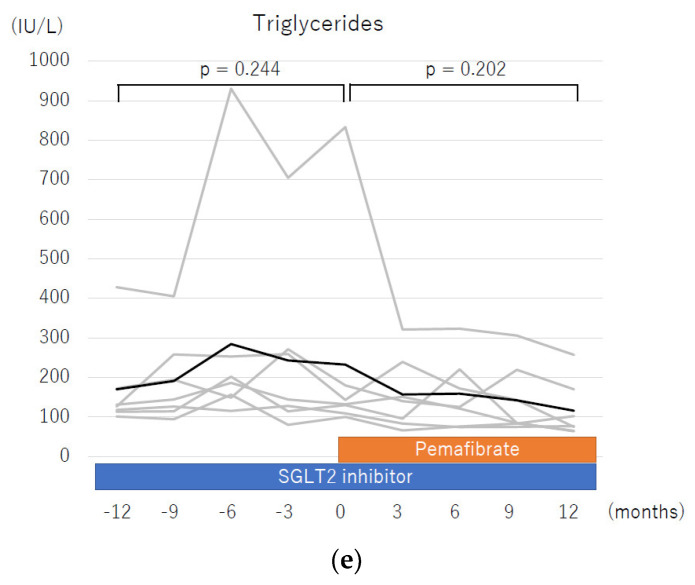
Changes in parameters during a two-year treatment course including one year of sodium glucose cotransporter-2 (SGLT2) inhibitor therapy and one year of pemafibrate add-on therapy. The changes in each parameter shown include mean value of (**a**) weight, (**b**) aspartate aminotransferase, (**c**) alanine aminotransferase, (**d**) γ-glutamyl transpeptidase, and (**e**) serum triglycerides. All statistical analyses were performed using Friedman’s test. Dark black line: mean; grey line: each patient.

The two-year changes in weight, AST, ALT, γ-GTP, and serum triglyceride before and after starting pemafibrate therapy are shown in Figure 2. Weight did not significantly change for two years. The serum AST, ALT, and γ-GTP levels had not significantly changed for one year before starting pemafibrate. Therefore, these parameters were surely refractory to long-term SGLT2 inhibitor therapy. After starting pemafibrate, the serum AST, ALT, and γ-GTP levels reduced significantly just after starting pemafibrate therapy and were maintained at reduced levels.

## 4. Discussion

This long-term observational pilot study showed that one year of pemafibrate therapy significantly improved markers of hepatic inflammation (ALT), function (ALBI score), and fibrosis (M2BPGi) in patients with both T2DM and NAFLD who were refractory to long-term therapy using SGLT2 inhibitors. In addition, these favorable outcomes were noted without significant changes in body weight or glycemic control, probably by a different mechanism. Thus, pemafibrate therapy is an option for the treatment of patients with NAFLD refractory to SGLT2 inhibitor therapy.

SGLT2 inhibitors are one choice to treat patients with both T2DM and NAFLD. Komiya et al. reported that ipragliflozin improved liver enzyme levels regardless of weight reduction in patients with NAFLD complicated by T2DM [17]. In addition, Ohki et al. reported that SGLT2 inhibitor was effective for patients with NAFLD refractory to incretin-based therapies, including glucagon-like peptide-1 (GLP-1) analogues and dipeptidyl peptidase-4 (DPP-4) inhibitors. Of 24 patients, 14 patients (58%) had normalization of serum ALT levels (median administration period: 320 days) [18]. Other studies also support that SGLT inhibitors can improve hepatic inflammation [19,20]. However, most of these studies failed to show a decrease in serum lipid levels, including triglyceride. In our previous study, serum triglyceride did not decrease despite a significant improvement in other metabolic and hepatic parameters, including glucose and liver enzyme levels [7]. Thus, other treatment options are needed to treat patients with NAFLD who have T2DM and dyslipidemia, common complications of NAFLD.

We previously reported that pemafibrate improved hepatic inflammation, function, and fibrosis in patients with NAFLD complicated by dyslipidemia [9,10,11]. At that time, we excluded patients with T2DM to reduce bias due to insulin resistance. Currently, little information is available on the effect of pemafibrate in patients with both NAFLD and T2DM. In addition, pemafibrate improved hepatic inflammation in NAFLD mice [21]. In the present study, the beneficial effects of pemafibrate on inflammation, function, and fibrosis were reproduced even in patients with both NAFLD and T2DM. These findings are in agreement with the placebo-controlled phase II trial of pemafibrate for patients with NAFLD [22]. In addition, the present study also demonstrated an improvement in liver function assessed by ALBI score, which is frequently used for decision making in the treatment of patients with HCC. Thus, the present study provides new information regarding the use of pemafibrate for patients with both NAFLD and T2DM.

The mechanism by which pemafibrate improves hepatic inflammation may differ from that induced by SGLT2 inhibitors. Pemafibrate, the selective and potent synthetic agonist of the PPAR-alpha nuclear receptor, was made available in Japan in 2018. It increases the activity of lipoprotein lipase that hydrolytes VLDL cholesterol and chylomicron, resulting in reduced plasma triglyceride levels. Furthermore, it also reduces hepatic VLDL cholesterol and triglyceride production [8]. In detailed analysis of lipoprotein profiles, pemafibrate increased apolipoprotein A-I [23], which may elevate HDL-cholesterol. However, a PPAR-alpha agonist also increased fractional catabolic rate, resulting in small changes in serum HDL-cholesterol levels. In addition, pemafibrate had small changes in low-density lipoprotein (LDL)-apoB-100 kinetics [24]. Thus, effects of pemafibrate on HDL-cholesterol and LDL-cholesterol were limited. This improvement in hepatic lipid profiles may explain the results of the PROMINET trial that pemafibrate significantly reduced the incidence of NAFLD in patients with T2DM, mild-to-moderate hypertriglyceridemia, low levels of HDL cholesterol, and well-controlled levels of LDL cholesterol [8]. In the present study, pemafibrate improved hepatic inflammation without significant changes in body weight. A Japanese double-blind, placebo-controlled phase II RCT of pemafibrate 0.1 mg twice daily for 72 weeks reported that pemafibrate significantly improved liver enzyme levels without changing body weight. In addition, pemafibrate did not reduce total liver fat volume compared to that of the control group evaluated by magnetic resonance imaging-derived proton density fat fraction (MRI-PDFF) [22]. These results of the phase II trial can be explained by the effect of pemafibrate on lipid droplets in the liver. Pemafibrate reduces the size of lipid droplets but does not reduce the total amount of liver fat [21]. Therefore, the primary endpoint of the next trial should be improvement in fibrosis and/or inflammation but not total amount of liver fat.

The strong anti-inflammatory effect of PPAR-alpha on the obesity-induced steatohepatitis has been reported in animal experiments [25]. PPAR-alpha knockout mice exposed to a chronic high-fat diet increased inflammation in hepatic and adipose tissue accompanied with increased chemokines and macrophage markers compared to the low-fat diet model. These inflammatory markers in the liver were well-correlated with hepatic triglyceride, and they were downregulated by treatment with the PPAR-alpha ligand. Therefore, PPAR-alpha has a protection effect against obesity-induced chronic liver inflammation in the mice model [25], suggesting that PPAR-alpha plays a pivotal role in controlling hepatic inflammation and lipid metabolism. The effect of pemafibrate on NAFLD was reported using an NAFLD mouse model [21]. Pemafibrate improves macrophage accumulation, ballooning degeneration of hepatocytes without reducing hepatic triglyceride levels in a mouse model of NAFLD. Pemafibrate also prevents NAFLD development by reducing myeloid cell recruitment via interaction with liver sinusoidal endothelial cells. An increased number and reduced size of lipid droplets in the liver is a main mechanism of pemafibrate [21]. A subsequent study with an experimental mouse model also revealed that pemafibrate reduces the size of lipid droplets and increases the number of lipid droplets [12], which may contribute to a decreased inflammatory reaction.

In contrast to pemafibrate, SGLT2 inhibitor treatment reduces body weight and total hepatic fat volume among “responders” [26]. The major mechanism by which SGLT2 inhibitors improve diabetic markers is by inhibition of glucose reabsorption in the proximal convoluted tubules in the kidney, leading to plasma glucose reduction and weight loss due to urinary calorie loss. As a result, SGLT2 inhibitors can reduce hepatic fat volume. Sumida et al. reported a significant reduction in hepatic fat content after 24 weeks’ treatment assessed by MRI. In addition, a reduction in hepatic fat content significantly correlated with the reduction in ALT level [19]. Furthermore, Kahl et al. also reported that empagliflozin treatment showed a significant reduction in liver fat content evaluated by MRI in 84 patients with NAFLD and well-controlled T2DM in an RCT [20]. The E-LIFT trial from India reported that SGLT2 inhibitor therapy for 20 weeks reduced hepatic fat seen on MRI-PDFF [27]. The reduction in total fat caused by SGLT2 inhibitors as assessed by MRI-PDFF is also evident in a systematic review [28]. Reduction in hepatic fat may be associated with the loss of body weight noted with SGLT2 inhibitor treatment. In short, pemafibrate reduced size of lipid droplets in the liver without changing total liver fat, but SGLT2 inhibitor decreased total liver fat by promoting catabolism. This evidence suggests that pemafibrate and SGLT2 inhibitors have different mechanisms by which they improve NAFLD. It also suggests that the addition of pemafibrate to the treatment of patients with NAFLD refractory to SGLT2 inhibitor therapy may have an added benefit.

Although the present pilot study focused on the effect of pemafibrate in patients with both NAFLD and T2DM who were refractory to SGLT2 inhibitor therapy, the results suggest that the combination of pemafibrate and SGLT2 inhibitor is useful for patients with NAFLD who suffer from both T2DM and dyslipidemia. Combination therapy significantly decreased hepatic steatosis/fibrosis in an experimental NAFLD mouse model [12]. In addition, combination therapy delayed the development of hepatocellular carcinoma, resulting in better survival compared to the control group. A case report supported the use of combination therapy with pemafibrate and an SGLT2 inhibitor and the three months of combined treatment improved hepatic inflammation and steatosis [29]. Thus, we believe that combination therapy may prevent progression to cirrhosis or hepatocellular carcinoma by suppressing persistent hepatic inflammation. Future RCTs evaluating the effect of pemafibrate in patients with NAFLD should focus on improvements in hepatic fibrosis rather than total amount of liver fat. Currently, an RCT (ClinicalTrials.gov Identifier: NCT05327127) is being undertaken to assess the effects of pemafibrate and/or an SGLT2 inhibitor on patients with NAFLD.

There are potential combination therapies for patients with triple comorbidities, including NAFLD, T2DM, and dyslipidemia. Although the first-line medication for patients with T2DM is metformin regardless of the presence of NAFLD, there is a lack of evidence for using metformin for patients with NAFLD in the clinical setting. Metformin inhibits mitochondrial respiratory chain and adenosine triphosphate (ATP) synthesis leading to increased intracellular adenosine monophosphate (AMP)/ATP ratio. Then, enhanced AMP-activated protein kinase suppresses gluconeogenesis and promotes lipid metabolism and beta-oxidization. Despite the favorable effects of metformin on hepatic lipogenesis in animal models of NAFLD, there is no rigorous evidence in clinical studies [30]. An RCT showed that the metformin group had a greater improvement in fatty liver as determined by sonography grade and serum liver enzyme levels in comparison to the pioglitazone group. However, all patients in both groups had vitamin E at a dose of 800 IU daily for six months [31], suggesting that the additional effects of vitamin E are not necessarily ruled out. A comparative study between metformin monotherapy and as a combination therapy (metformin + empagliflozin) in patients with T2DM and NAFLD showed superiority of the latter group for improvements in ALT and hepatic fibrosis, and therefore the degree of amelioration of SGLT2 inhibitor may be greater than that of metformin [32]. As a result, Japanese evidence-based clinical practice guidelines for NAFLD do not recommend metformin for the specific treatment of NAFLD due to a lack of evidence suggesting improvement in serum liver enzyme levels and liver histology [5]. Although three patients included in the present study were treated with metformin before starting pemafibrate therapy, the use of metformin may not provide a large influence on the results of the present study.

Statins (HMG-CoA reductase inhibitor) are considered the gold standard treatment for patients with dyslipidemia worldwide, and statin use remarkably reduces the risk of cardiovascular and cerebrovascular complications. Despite the lack of high-level evidence regarding the effect of statins on patients with NAFLD, the use of statins is weakly suggested by the Japanese guidelines for the treatment of patients with NAFLD [5]. In a systematic review, statins improved serum AST and ALT levels in patients with NAFLD compared to the control group [33]. A large European study reported the dose-dependent effect of statins on hepatic steatosis and fibrosis [34]. A prospective study including 20 patients with biopsy-proven NAFLD showed that rosuvastatin therapy (10 mg/day) for one year normalized liver enzymes in 19 patients without significant weight reduction [35]. A second biopsy performed in three patients showed improvement of hepatocyte ballooning degeneration, lobular inflammation, and fibrosis [35]. In a mouse model of diet-induced obesity, rosuvastatin therapy reduced hepatic triglycerides compared to the control group in a dose-dependent manner and changed the fat distribution from visceral to subcutaneous [36]. A long-term clinical study (follow-up period: 10.3–16.3 years) reported a significant reduction in liver steatosis in patients treated with statins compared to those treated without statins [37]. A prospective study including 31 patients with biopsy-proven NAFLD complicated with hyperlipidemia showed that atorvastatin therapy (10 mg daily for 24 months) improved adiponectin, tumor necrosis factor-alpha, transaminase, LDL cholesterol, type IV collagen, and CT liver–spleen ratio. In addition, atorvastatin therapy improved steatosis grade on histologic assessment [38]. Although two patients included in the present study received statin therapy, the statin therapy started more than one year before starting pemafibrate, and therefore the influence of statin therapy is small.

GLP-1 analogue is a candidate for NAFLD therapy complicated by T2DM, and it is suggested in the treatment guidelines for NAFLD [5]. Incretin is secreted by the small intestine after meals and increases insulin secretion and sensitivity. The main incretins are GLP-1 and glucose-dependent insulinotropic polypeptide (GIP). GLP-1 decreases bowel movements, resulting in enhanced gastric retention and early satiety leading to effective weight reduction. Liraglutide, a once daily GLP-1 agonist, improves glycemic control by ameliorating the function of beta-cells. Ohki et al. reported the effectiveness of liraglutide for the treatment of patients with NAFLD, and the liraglutide group showed significant weight and ALT reduction [39]. A double-blind RCT in the United Kingdom revealed that liraglutide (1.8 mg daily) for 48 weeks resulted in significant histological resolution of NAFLD compared to placebo. The resolution rate in the NAFLD group was significantly higher (39%) compared to that of the placebo group (9%), but the RCT also demonstrated significant weight reduction in the liraglutide group [40]. Shao et al. compared the effect of exenatide with intensive insulin, and the exenatide group showed significantly better hepatic profiles accompanied with weight reduction compared to the intensive insulin group [41]. A Japanese cohort demonstrated that liraglutide (0.9 mg once daily) for 24 weeks significantly improved weight, visceral fat accumulation, and transaminase levels in 19 patients with NAFLD and T2DM. Histological changes before and after treatment revealed a decrease in histological inflammation in 8 of 10 patients who underwent follow-up liver biopsy at 96 weeks [42]. A prospective study from the United Kingdom reported that a GLP-1 agonist (exenatide or liraglutide) for six months was significantly associated with 5% weight reduction and 42% intrahepatic lipid reduction evaluated by MRI [43]. Since the presence of a GLP-1 receptor on the surface of hepatocytes is not clearly proven, hepatic improvements due to GLP-1 agonists may largely be affected by weight reduction. Obesity is one of the most important factors in the pathogenesis of NAFLD, and the effectiveness of weight reduction on NAFLD is undoubtedly evident. One patient in the present study had concurrent GLP-1 treatment for two years before starting pemafibrate therapy, suggesting that the combination of a SGLT2 inhibitor and pemafibrate therapy may be effective therapy for patients refractory to GLP-1. Unlike most GLP-1 or SGLT2 inhibitor studies evaluating the effect on NAFLD, pemafibrate improves NAFLD without changing weight.

DPP-4 is a serine protease enzyme inducing inactivation of incretin hormones. DPP-4 inhibitors can be administered orally and efficiently suppress the activity of DPP-4, resulting in increased GLP-1 levels but decreased glucagon levels in the circulation. DPP-4 is expressed in the liver, and serum DPP-4 activity is elevated in NAFLD subjects compared to healthy controls [44]. An RCT showed that sitagliptin (100 mg daily) for 24 weeks did not improve serum liver enzyme levels or fibrosis in 12 patients with biopsy-proven NAFLD compared to the placebo group. There were no significant changes in adiponectin level or liver steatosis as evaluated by MRI [45]. Unlike the effectiveness of GLP-1 agonist studies on NAFLD, most studies showed that a DPP-4 inhibitor led to little improvement in patients with diabetes and NAFLD [46]. The present study included three patients treated with DPP-4 inhibitors, but combined use of DPP-4 inhibitors might not influence these results.

This study has acknowledged limitations. First, this is a retrospective observational pilot study including a small number of patients, but significant improvement in multiple hepatic marker levels was observed. Second, combined treatment such as the added use of statins might introduce bias in the results. Third, there were no histological evaluations of hepatic tissue. The major advantage of this study is a long-term follow-up period of two years. Patients without one-year follow-up after starting pemafibrate or one year of treatment with an SGLT2 inhibitor before starting pemafibrate were excluded. Based on the one-year clinical course of patients treated with SGLT2 inhibitors, the initiation effect of prior SGLT2 inhibitor was likely negligible. Furthermore, no weight reduction was observed during one year of pemafibrate therapy, and therefore the improvements observed were most likely a result of the pemafibrate treatment. Even with long-term treatment using pemafibrate, a recent RCT demonstrated that there were no differences in adverse events or renal function during the 72-week study period comparing the pemafibrate and placebo groups [22].

## 5. Conclusions

One year of pemafibrate therapy improves hepatic inflammation, function, and fibrosis without changing weight or glycemic control in patients with NAFLD refractory to long-term SGLT2 inhibitor therapy. Pemafibrate, a medication that targets lipid metabolism, can improve NAFLD in patients who are not responding to long-term treatment with SGLT2 inhibitors. This finding is significant because it provides a potential treatment option for patients with NAFLD who do not respond to current therapies. Additionally, this study highlights the need for continued research into effective treatments for NAFLD, a prevalent and serious condition with limited treatment options. To the best of our knowledge, this is the first original report to evaluate the effect of the addition of pemafibrate to treatment with SGLT2 inhibitors in patients with NAFLD. The results of this pilot study are encouraging and support the conduct of an RCT to confirm these preliminary results.

## Figures and Tables

**Table 1:** Baseline characteristics.

	*n* = 7
Age, years, median (range)	49 (42–68)
Gender, male, *n*	7 (100%)
Current smoker, *n*	1 (14%)
Complications treated with medications, *n*	
Hypertension	4 (57%)
Gastroesophageal reflux disease	5 (71%)
Diabetes mellitus	7 (100%)
Concurrent medications, *n*	
SGLT2 inhibitor	7 (100%)
Metformin	3 (43%)
Dipeptidyl peptidase 4 inhibitor	3 (43%)
Statins	2 (29%)
Glucagon-like peptide-1 receptor agonist	1 (14%)
Angiotensin II receptor blocker	0 (0%)
Interval from starting SGLT2 inhibitor to starting pemafibrate, day, median (range)	845 (406–1288)

SGLT2: sodium glucose cotransporter-2.

**Table 2:** Changes in clinical parameters after one year of pemafibrate therapy.

	Baseline	One Year Later	*p*-Value	Reference Value
Weight, kg, median (range)	73.3 (66.2–112.1)	74.6 (64.6–114.8)	0.937	-
Body mass index	28.3 (22.2–37.4)	27.8 (21.7–38.3)	1.000	-
AST, U/L	65 (31–130)	29 (25–44)	0.015	10–40
ALT, U/L	86 (33–175)	33 (19–47)	0.015	5–45
γ-GTP, U/L	80 (41–173)	43 (27–73)	0.015	M ≦ 80 F ≦30
Platelet count, ×10^4^/μL	22.9 (11.1–31.9)	22.5 (13.2–26.5)	0.937	14.0–34.0
Estimated GFR, mL/min/1.73 m^2^	73.9 (63.8–89.7)	64.1 (54.3–92.9)	0.296	-
LDL cholesterol, mg/dL	122 (103–190)	114 (64–169)	0.132	65–139
HDL cholesterol, mg/dL	45 (30–62)	51 (34–65)	0.156	M 40–85 F 40–95
Triglyceride, mg/dL	132 (100–833)	77 (64–257)	0.031	30–149
Uric acid, mg/dL	5.9 (3.8–7)	5.9 (4.2–6.8)	0.687	M 3.8–7.0 F 2.5–7.0
Fasting plasma glucose, mg/dL	130 (100–198)	112 (94–215)	0.578	<110
Serum insulin, µU/mL	9.8 (1.2–14)	5.5 (2.6–47.6)	1.000	1.7–10.4
Hemoglobin A1c, %	6.8 (6.2–7.6)	6.5 (6.2–7.8)	0.765	4.6–6.2
Total bilirubin, mg/dL	1.0 (0.4–1.5)	1.0 (0.3–1.3)	0.218	0.2–1.2
Serum albumin, g/dL	4.4 (4.2–4.6)	4.5 (4.2–4.9)	0.250	3.8–5.2
ALBI score	−2.9 (−3.1–2.7)	−3.1 (−3.4–2.8)	0.031	≦−2.6
M2BPGi	0.73 (0.4–2.32)	0.62 (0.34–1.66)	0.046	<1.00

AST: aspartate aminotransferase, ALT: alanine aminotransferase, γ-GTP: γ-glutamyl transpeptidase, GFR: glomerular filtration rate, LDL: low-density lipoprotein, HDL: high-density lipoprotein, ALBI: Albumin-Bilirubin, M2BPGi: Mac-2 Binding Protein Glucosylation Isomer, M: male, F: female.

## Data Availability

The datasets generated during and/or analyzed during the current study are available from the corresponding author on reasonable request.
